# The Mental Health of Incarcerated Immigrants, Survey of Prison Inmates, 2016

**DOI:** 10.1007/s10903-025-01749-z

**Published:** 2025-07-29

**Authors:** Rachel A. Zajdel, Chelsea Duong, Erik J. Rodriquez, Eliseo J. Pérez-Stable

**Affiliations:** Division of Intramural Research, National Heart, Lung, and Blood Institute, National Institutes of Health, Bethesda, United States

**Keywords:** Immigrant health advantage, Mental health, Incarceration, Race and ethnicity

## Abstract

Immigrants tend to exhibit better mental health compared to their U.S.-born counterparts, but this immigrant health advantage is not uniform across immigrant populations. The objective of this study is to examine if the immigrant health advantage extends to incarcerated individuals and if there are differences by race and/or ethnicity. We used data from the 2016 Survey of Prison Inmates (n=20,226) and multivariable linear and logistic regression models to examine the relationship between immigration status, race and/or ethnicity, and mental health (psychological distress, depression, anxiety) among imprisoned individuals. The analytic sample included Black (n=7,104), Latino (n=5,029), and White (n=8,093) respondents, ten percent of whom were foreign-born. The average Kessler 6-item psychological distress score was 5.9 and 24.4% and 19.8% of respondents reported ever having been diagnosed with depression and anxiety, respectively. Incarcerated U.S.-born individuals reported significantly more symptoms of psychological distress (**β**=0.89; C.I.=0.46,1.32; p<0.001) and higher odds of depression (OR=2.03; C.I.=1.64,2.50; p<0.001) and anxiety (OR=2.30; C.I.=1.82, 2.90; p<0.001) compared to incarcerated foreign-born individuals. When stratified by race and/or ethnicity, the same patterns emerged for incarcerated Latino individuals by U.S. birth. In contrast, incarcerated U.S.-born Black individuals had lower levels of psychological distress (**β**=–1.64; C.I.=–2.99,–0.29; p<0.05) compared to incarcerated foreign-born Black individuals. No relationship between immigration status and mental health was observed among incarcerated White individuals. Findings suggest that the immigrant health advantage may vary by race and ethnicity within the imprisoned population.

**Supplementary Information:**

The online version contains supplementary material available at 10.1007/s10903-025-01749-z.

## Background

Foreign-born individuals tend to exhibit better mental health than U.S.-born individuals [1, 2]. For example, Black, Latino, and White immigrants display less symptoms of psychological distress relative to their U.S.-born counterparts [1, 3]. Research suggests this immigrant health advantage is produced by overlapping mechanisms including the self-selection of healthier individuals into migration [4] and social support embedded in migrant networks [5]. However, the immigrant health advantage in mental health tends to decline with increased time in the U.S [2].

One possible health eroding factor that may affect immigrants’ mental health is exposure to the criminal legal system. More specifically, the experience of incarceration could separate immigrants from social support and lead to poor mental well-being. The removal of this social support may be especially impactful for Black and Latino immigrants due to existing inequities in incarceration. Black and/or Latino individuals are at significantly higher risk of imprisonment than White individuals [6]. The U.S. criminal legal system also uniquely targets and punishes unauthorized immigrants, as well as residents who are non-U.S. citizens [7], which contributes to racial and/or ethnic disparities in imprisonment [8].

Confinement in prisons is associated with poor mental well-being [9], even after release [10]. While these findings extend to immigrants held in detention facilities [11], the mental health of immigrants currently incarcerated in correctional facilities has not been studied. To address this gap, the present study is the first to examine if the immigrant health advantage in mental health extends to incarcerated persons and if there are differences by race and/or ethnicity. Findings highlight unexplored inequalities in health among incarcerated individuals.

## Data and Methods

Data came from the Survey of Prison Inmates (SPI), a nationally representative sample of adults held in state and federal prisons. The SPI was most recently conducted by the Bureau of Justice Statistics in January-October 2016 through face-to-face interviews using computer-assisted personal interviewing. The SPI gathers information about demographic characteristics, family background, health, and criminal offenses. The response rate was 69.3% and 72.8% for individuals held in state and federal correctional facilities, respectively [12]. In the present study, we used deidentified public-use data, which is distributed online through the University of Michigan Inter-University Consortium for Political and Social Research (ICPSR) and does not necessitate IRB approval.

### Dependent Variables

Mental health included symptoms of psychological distress and self-reported physician-diagnosed conditions. *Psychological distress* was measured using the Kessler six-item (K6) past 30-day non-specific distress scale [13]. Respondents were asked how often they felt nervous, hopeless, restless or fidgety, depressed, that everything was an effort, and worthless. Answers included “none of the time” (0), “a little of the time” (1), “some of the time” (2), “most of the time” (3), and “all of the time” (4) and were summed to generate a score between 0 and 24. A score of ≥ 13 has been established as an accurate indicator of meeting DSM-IV criteria for a mental illness [14].

We examined two mental health conditions—depression and anxiety—given that their frequency of diagnosis allowed for analyses at the intersection of immigration status and race and/or ethnicity. *Depression* was measured with the question, “Have you ever been told by a medical doctor or mental health professional, such as a psychiatrist or psychologist, that you had a depressive disorder?” (1 = yes). *Anxiety* was assessed with a similar question as to whether an individual had ever been diagnosed with “an anxiety disorder, such as panic disorder or obsessive-compulsive disorder, also known as OCD” (1 = yes).

### Independent Variables

*Race* was measured as an individual’s self-identified race and/or ethnicity: Black or African American (*n* = 7,104), Latino or Hispanic (*n* = 5,029), and White (*n* = 8,093). White individuals served as the reference due to their higher socioeconomic status in the U.S. Other racial groups were excluded due to small sample sizes once stratified by immigration status.

Immigration status was measured as *U.S. birth*, defined as whether an individual was born in the U.S. or its territories (1) or in another country (0). In supplemental models, we also assessed if health outcomes varied by *U.S. citizenship* (1 = U.S. citizen; 0 = not), regardless of U.S birth. We chose to model U.S. birth and U.S. citizenship separately due to a high degree of correlation between the variables (*r* =.81).

### Covariates

Multivariable models included available sociodemographic variables associated with health. *Sex* was a dichotomous variable (1 = female). *Age* was a categorical variable: 18–34 (reference), 35–49, and ≥ 50 years. *Education* measured respondents’ highest level of education prior to incarceration: less than high school (reference), high school degree, some college or Associate’s degree, and college degree or more. *Married* indicated if a person was married (1) or not (0). *Health insurance* measured if a respondent had health insurance 30 days prior to arrest (1) or not (0). C*rime type* indicated the primary offense category of which a respondent was convicted: violent (reference), property, drug, or public order. In supplemental analyses, we included length of U.S. residence (in years) to evaluate its potential influence on mental health. For models including U.S.-born individuals, their age (in years) was used.

### Analytic Strategy

First, we assessed the association between U.S. birth and mental health. To do this, we used bivariate and multivariable linear regression for the psychological distress models and bivariate and multivariable logistic regression for the depression and anxiety models. Results are presented as coefficients for linear regressions and odds ratios for logistic regressions, with 95% confidence intervals.

Second, we examined if the association between U.S. birth and mental health differed by race and/or ethnicity using race-stratified bivariate and multivariable linear and logistic regressions. Results using interaction terms were substantively similar. We therefore chose to present stratified models given their relative ease of interpretation compared to interaction terms.

The analytic sample (*n* = 20,226) excluded individuals who identified as anything other than Black, Latino, or White (*n* = 3,251), had an unknown crime type (*n* = 110), or had missing data for any of the examined variables (*n* = 610). We utilized survey and replicated weights using the *svyset* command and jackknife method of variance estimation, as outlined in the SPI User Guide [15]. This accounted for survey design, nonresponse, and post-stratification adjustment and thereby produced national estimates of the U.S. prison population. All analyses were conducted in Stata-17.

## Results

A plurality of respondents identified as Black (38.8%), followed by White (35.6%) and Latino (25.7%) (Table 1). Foreign-born persons were 10.6% of the sample and most immigrants were Latino (85.3%). Incarcerated U.S.-born individuals exhibited more symptoms of psychological distress than incarcerated foreign-born individuals (6.1 versus 4.7; *p* <.001). They also reported more than double the prevalence diagnoses of depression (25.7%) and anxiety (20.9%) disorders compared to incarcerated foreign-born individuals (12.7% and 10.0%; *p* <.001).

**Table 1:** Weighted descriptive statistics for respondents to the 2016 survey of prison inmates, stratified by birthplace

	U.S.-born (*n* = 18,083)	Foreign-born (*n* = 2,143)	Total (*n* = 20,226)
*Demographic Characteristics*			
Female (%)	4,791 (7.2%)	272 (3.7%)***	5,063 (6.9%)
Age category (%)			
18–34 years	7,594 (42.3%)	726 (36.0%)***	8,320 (41.6%)
35–49 years	6,859 (37.1%)	955 (42.3%)***	7,814 (37.6%)
50 + years	3,630 (20.6%)	462 (21.7%)	4,092 (20.7%)
Education (%)			
Less than high school	10,436 (61.6%)	1,366 (66.2%)**	11,802 (62.1%)
High school degree	4,237 (22.8%)	418 (19.4%)**	4,655 (22.4%)
Some college or Associate’s	2,475 (11.7%)	204 (8.6%)***	2,679 (11.4%)
College degree or more	935 (3.9%)	155 (5.8%)**	1,090 (4.1%)
Race and/or Ethnicity (%)			
Black	6,953 (42.3%)	151 (7.4%)***	7,104 (38.8%)
Latino	3,195 (19.0%)	1,834 (85.3%)***	5,029 (25.7%)
White	7,935 (38.7%)	158 (7.3%)***	8,093 (35.6%)
Married (%)	2,648 (13.9%)	576 (25.6%)***	3,224 (15.0%)
Health insurance (%)	5,307 (29.9%)	554 (25.6%)***	5,861 (29.5%)
U.S. Citizen (%)	18,083 (100.0%)	643 (30.1%)***	18,726 (93.0%)
Length of U.S. residence^a^ (mean, se)	-	22.2 (0.7)	-
*Incarceration Characteristics*			
Crime type (%)			
Violent	8,296 (51.3%)	677 (41.5%)**	8,973 (50.3%)
Property	3,246 (15.3%)	184 (6.5%)***	3,430 (14.5%)
Drug	3,779 (18.5%)	693 (29.7%)***	4,472 (19.6%)
Public order	2,762 (14.9%)	589 (22.4%)***	3,351 (15.7%)
*Mental Health Outcomes*			
Psychological distress (mean, se)	6.1 (0.1)	4.7 (0.2)***	5.9 (0.1)
Depression (%, yes)	5,456 (25.7%)	268 (12.7%)***	5,724 (24.4%)
Anxiety (%, yes)	4,673 (20.9%)	208 (10.0%)***	4,881 (19.8%)

^a^ Within foreign-born individuals only.

*p <.05; **p <.01; ***p <.001 statistically significant difference from U.S.-born respondents.

Incarcerated foreign-born Black, Latino, and White respondents tended to have higher levels of education than their U.S.-born counterparts (Table 2). Black and Latino immigrants were also significantly more likely to be married, while Latino and White immigrants tended to be older and less likely to be female compared to the U.S.-born. Latino immigrants were less likely to be convicted of a violent offense and White immigrants were more likely to have had health insurance prior to incarceration than their U.S.-born counterparts.

**Table 2:** Weighted descriptive statistics for respondents to the 2016 survey of prison inmates, stratified by race and/or ethnicity and birthplace

	Black		Latino		White	
	U.S.-born (*n* = 6,953)	Foreign-born (*n* = 151)	U.S.-born (*n* = 3,195)	Foreign-born (*n* = 1,834)	U.S.-born (*n* = 7,935)	Foreign-born (*n* = 158)
*Demographic Characteristics*						
Female (%)	1,098 (4.2%)	18 (3.4%)	707 (6.9%)	213 (3.6%)***	2,986 (10.8%)	41 (5.7%)***
Age category (%)						
18–34 years	2,952 (43.5%)	50 (34.7%)	1,679 (52.0%)	631 (36.2%)***	2,963 (36.2%)	45 (34.1%)
35–49 years	2,654 (37.5%)	65 (42.8%)	1,132 (35.5%)	840 (43.6%)***	3,073 (37.4%)	50 (27.3%)*
50 + years	1,347 (19.0%)	36 (22.5%)	384 (12.5%)	363 (20.2%)***	1,899 (26.4%)	63 (38.6%)*
Education (%)						
Less than high school	4,529 (67.9%)	63 (46.4%)***	2,107 (68.4%)	1,265 (71.2%)	3,800 (51.3%)	38 (27.2%)***
High school degree	1,505 (20.9%)	35 (22.3%)	664 (20.8%)	339 (18.3%)	2,068 (25.8%)	44 (29.6%)
Some college or Associate’s	709 (8.9%)	33 (20.2%)**	323 (8.5%)	131 (6.3%)*	1,443 (16.4%)	40 (24.5%)
College degree or more	210 (2.3%)	20 (11.1%)**	101 (2.3%)	99 (4.2%)**	624 (6.5%)	36 (18.7%)***
Married (%)	859 (12.2%)	35 (22.6%)*	492 (15.4%)	500 (26.5%)***	1,297 (14.9%)	41 (18.8%)
Health insurance (%)	2,157 (30.9%)	46 (32.7%)	825 (26.3%)	428 (23.1%)	2,325 (30.6%)	80 (47.2%)***
U.S. citizen (%)	6,953 (100.0%)	60 (44.3%)***	3,195 (100.0%)	481 (26.0%)***	7,935 (100.0%)	102 (62.6%)***
Length of U.S. residence^a^ (mean, se)	-	25.6 (1.2)	-	21.1 (0.6)	-	31.1 (1.5)
*Incarceration Reason*						
Crime type (%)						
Violent	3,700 (55.9%)	70 (55.9%)	1,489 (52.3%)	552 (40.1%)**	3,107 (45.7%)	55 (43.1%)
Property	902 (11.3%)	21 (9.5%)	497 (14.1%)	113 (4.8%)***	1,847 (20.4%)	50 (23.1%)
Drug	1,382 (19.4%)	32 (19.9%)	728 (18.5%)	642 (31.8%)***	1,669 (17.4%)	19 (15.0%)
Public order	969 (13.4%)	28 (14.6%)	481 (15.1%)	527 (23.4%)**	1,312 (16.5%)	34 (18.8%)
*Mental Health Outcomes*						
Psychological distress (mean, se)	5.9 (0.1)	7.2 (0.7)	6.0 (0.2)	4.4 (0.2)***	6.4 (0.1)	5.7 (0.6)
Depression (%, yes)	1,436 (18.9%)	27 (18.5%)	924 (25.0%)	188 (10.3%)***	3,096 (33.5%)	53 (34.7%)
Anxiety (%, yes)	941 (12.3%)	16 (11.5%)	838 (22.5%)	157 (8.4%)***	2,894 (26.7.3%)	35 (27.1%)

^a^ Within foreign-born individuals only.

*p <.05; **p <.01; ***p <.001 statistically significant difference from U.S.-born respondents

### Immigration Status and Mental Health

Bivariate models showed a significant positive relationship between U.S. birth and each of the mental health outcomes in the total sample, a pattern driven by Latino individuals (Table 3). This relationship remained significant upon including sociodemographic variables, with incarcerated U.S.-born individuals reporting significantly more symptoms of psychological distress relative to incarcerated foreign-born individuals (**β** = 0.89; C.I.=0.46, 1.32; *p* <.001). They also exhibited over two times higher odds of reporting depression (OR = 2.03; C.I.=1.64, 2.50; *p* <.001) or anxiety (OR = 2.30; C.I.=1.82,2.90; *p* <.001) compared to their foreign-born counterparts.

**Table 3:** Immigration status and mental health outcomes regression models, 2016 survey of prison inmates (*n* = 20,226)

	Model 1: Psychological Distress		Model 2: Depression		Model 3: Anxiety	
	Coefficient	[95% C.I.]	Odds ratio	[95% C.I.]	Odds ratio	[95% C.I.]
*Bivariate*						
U.S.-born	1.35***	[0.91, 1.78]	2.38***	[1.92, 2.96]	2.39***	[1.91, 2.99]
*Multivariable*						
U.S.-born	0.89***	[0.46, 1.32]	2.03***	[1.64, 2.50]	2.30***	[1.82, 2.90]
Female	1.44***	[1.14, 1.75]	2.62***	[2.30, 2.99]	3.02***	[2.65, 3.43]
Age (ref = 18–34 years)						
35–49 years	−0.08	[−0.29, 0.14]	1.07	[0.96, 1.18]	1.00	[0.91, 1.11]
50 + years	−0.63***	[−0.93, −0.33]	0.82**	[0.72, 0.94]	0.69***	[0.60, 0.78]
Education (ref = < HS)						
High school	−0.49***	[−0.69, −0.29]	0.80***	[0.71, 0.90]	0.91	[0.81, 1.03]
Some college	−0.43**	[−0.71, −0.15]	0.94	[0.81, 1.10]	1.08	[0.94, 1.23]
College degree or more	−0.56*	[−1.01, −0.11]	0.99	[0.80, 1.22]	1.04	[0.81, 1.33]
Race and/or Ethnicity (ref = White)						
Black	−0.55***	[−0.78, −0.32]	0.47***	[0.42, 0.52]	0.35***	[0.31, 0.40]
Latino	−0.73***	[−1.07, −0.39]	0.60***	[0.52, 0.68]	0.64***	[0.55, 0.74]
Married	−0.04	[−0.29, 0.21]	0.93	[0.83, 1.04]	1.02	[0.91, 1.14]
Health insurance	−0.84***	[−1.03, −0.66]	0.74***	[0.67, 0.81]	0.80***	[0.72, 0.89]
Crime type (ref = violent)						
Property	−0.30*	[−0.58, −0.02]	0.95	[0.85, 1.07]	1.10	[0.96, 1.27]
Drug	−1.12***	[−1.44, −0.81]	0.62***	[0.55, 0.72]	0.78***	[0.69, 0.88]
Public order	−0.72***	[−0.98, −0.46]	0.90	[0.80, 1.02]	1.07	[0.93, 1.22]

Author’s calculations using data from the 2016 Survey of Prison Inmates. Weighted statistics from linear regression (Model 1) and logistic regression (Models 2 and 3). *p <.05; **p <.01; ***p <.001

### Immigration Status, Race and/or Ethnicity, and Mental Health

As in the total incarcerated sample, bivariate and multivariable models demonstrated similar results among Latino individuals (Table 4). Imprisoned U.S.-born Latino individuals exhibited significantly more symptoms of psychological distress (**β** = 1.27; C.I.=0.81,1.73; *p* <.001) and greater odds of depression (**OR** = 2.57; C.I.=2.02,3.27; *p* <.001) and anxiety (**OR** = 2.92; C.I.=2.25,3.79; *p* <.001) relative to their foreign-born counterparts.

**Table 4:** Immigration status and mental health outcomes regression models, stratified by race and/or ethnicity, 2016 survey of prison inmates

		Model 1: Psychological Distress		Model 2: Depression		Model 3: Anxiety	
		Coefficient	[95% C.I.]	Odds ratio	[95% C.I.]	Odds ratio	[95% C.I.]
a. Black	*Bivariate*						
(*n* = 7,104)	U.S.-born	−1.32	[−2.75, 0.11]	1.03	[0.63, 1.68]	1.07	[0.50, 2.31]
	*Multivariable*						
	U.S.-born	−1.64*	[−2.99, −0.29]	0.93	[0.57, 1.53]	1.02	[0.46, 2.25]
	Female	1.69***	[1.25, 2.13]	2.94***	[2.37, 3.65]	3.13***	[2.47, 3.98]
	Age (ref = 18–34 years)						
	35–49 years	−0.06	[−0.38, 0.26]	1.14	[0.97, 1.34]	1.28**	[1.07, 1.52]
	50 + years	−0.84***	[−1.24, −0.44]	1.01	[0.82, 1.24]	0.93	[0.74, 1.18]
	Education (ref = < HS)						
	High school	−0.64***	[−0.97, −0.32]	0.61***	[0.51, 0.75]	0.78*	[0.63, 0.97]
	Some college	−0.94***	[−1.38, −0.50]	0.56***	[0.43, 0.72]	0.74	[0.54, 1.01]
	College degree or more	−1.35**	[−2.12, −0.58]	0.61	[0.32, 1.16]	0.78	[0.36, 1.69]
	Married	−0.12	[−0.55-0.31]	1.25*	[1.01–1.56]	1.13	[0.89–1.44]
	Health insurance	−0.89***	[−1.16, 0.62]	0.76**	[0.64, 0.90]	0.77*	[0.63, 0.95]
	Crime type (ref = violent)						
	Property	−0.20	[−0.67, 0.28]	1.10	[0.90, 1.34]	1.32*	[1.03, 1.70]
	Drug	−1.06***	[−1.43, −0.68]	0.64***	[0.52, 0.79]	0.63***	[0.49, 0.81]
	Public order	−0.47*	[−0.90, −0.04]	0.98	[0.77, 1.25]	1.31	[0.97, 1.77]
b. Latino	*Bivariate*						
(*n* = 5,029)	U.S.-born	1.52***	[1.05, 1.99]	2.90***	[2.27, 3.72]	3.17***	[2.47, 4.06]
	*Multivariable*						
	U.S.-born	1.27***	[0.81, 1.73]	2.57***	[2.02, 3.27]	2.92***	[2.25, 3.79]
	Female	2.10***	[1.45, 2.74]	2.94***	[2.28, 3.79]	3.36***	[2.66, 4.23]
	Age (ref = 18–34 years)						
	35–49 years	−0.15	[−0.63, 0.33]	1.02	[0.84, 1.22]	0.94	[0.76, 1.16]
	50 + years	−0.11	[−0.75, 0.52]	0.94	[0.70, 1.26]	0.73	[0.53, 1.01]
	Education (ref = < HS)						
	High school	−0.38	[−0.97, −0.32]	0.95	[0.76, 1.19]	0.93	[0.74, 1.16]
	Some college	0.03	[−0.61, 0.68]	1.30	[0.96, 1.76]	1.10	[0.79, 1.53]
	College degree or more	−0.67	[−1.58, 0.25]	1.31	[0.82, 2.07]	1.66*	[1.05, 2.64]
	Married	0.08	[−0.40-0.56]	0.90	[0.69–1.16]	1.05	[0.84–1.32]
	Health insurance	−0.74***	[−1.16, −0.33]	0.73**	[0.59, 0.91]	1.01	[0.82, 1.24]
	Crime type (ref = violent)						
	Property	−0.10	[−0.76, 0.58]	1.01	[0.76, 1.34]	1.12	[0.82, 1.53]
	Drug	−0.87**	[−1.44, −0.30]	0.55***	[0.43, 0.72]	0.81	[0.61, 1.08]
	Public order	−0.97***	[−1.48, −0.45]	0.81	[0.62, 1.05]	1.02	[0.77, 1.36]
c. White	*Bivariate*						
(*n* = 8,093)	U.S.-born	0.68	[−0.45, 1.81]	0.95	[0.64, 1.40]	1.14	[0.68, 1.91]
	*Multivariable*						
	U.S.-born	0.32	[−0.83, 1.48]	0.82	[0.55, 1.22]	0.99	[0.58, 1.70]
	Female	1.10***	[0.70, 1.49]	2.41***	[2.03, 2.85]	2.81***	[2.39, 3.30]
	Age (ref = 18–34 years)						
	35–49 years	−0.03	[−0.38, 0.32]	1.03	[0.89, 1.20]	0.90	[0.77, 1.05]
	50 + years	−0.69**	[−1.11, −0.27]	0.69***	[0.58, 0.83]	0.58***	[0.49, 0.68]
	Education (ref = < HS)						
	High school	−0.43*	[−0.80, −0.07]	0.87	[0.73, 1.04]	0.97	[0.82, 1.15]
	Some college	−0.34	[−0.77, 0.09]	1.07	[0.85, 1.36]	1.21*	[1.02, 1.44]
	College degree or more	−0.28	[−0.85, 0.28]	1.10	[0.84, 1.44]	1.04	[0.77, 1.40]
	Married	−0.02	[−0.40-0.37]	0.79**	[0.67–0.93]	0.97	[0.82–1.14]
	Health insurance	−0.88***	[−1.19, −0.57]	0.71**	[0.61, 0.82]	0.73***	[0.63, 0.85]
	Crime type (ref = violent)						
	Property	−0.50**	[−0.88, −0.12]	0.87	[0.73, 1.03]	1.01	[0.84, 1.21]
	Drug	−1.38***	[−1.92, −0.84]	0.65**	[0.51, 0.84]	0.84	[0.70, 1.03]
	Public order	−0.73**	[−1.15, −0.32]	0.93	[0.77, 1.11]	1.01	[0.85, 1.19]

Author’s calculations using data from the 2016 Survey of Prison Inmates. Weighted statistics from linear regression (Model 1) and logistic regression (Models 2 and 3). *p <.05; **p <.01; ***p <.001

There was no relationship between immigration status and mental health for incarcerated Black individuals in the bivariate models. However, upon controlling for sociodemographic variables, incarcerated U.S.-born Black individuals reported significantly lower levels of psychological distress (**β**=–1.64; C.I.= − 2.99, − 0.29; *p* <.05) relative to their foreign-born counterparts. This pattern did not extend to depression or anxiety. The mental health of incarcerated White individuals did not significantly differ by U.S. birth.

### Supplemental Analyses

Patterns were mostly similar when assessing U.S. citizenship instead of U.S. birth (Online Resource 1). Overall, 93% of incarcerated individuals, and 30.1% of incarcerated foreign-born individuals, were U.S. citizens. Incarcerated Latino individuals with U.S. citizenship exhibited significantly more symptoms of psychological distress and higher odds of depression and anxiety than incarcerated Latino individuals without U.S. citizenship. There was no significant relationship between naturalized citizenship status and psychological distress among incarcerated Black individuals, although there were only 91 non-U.S. citizens in this group (Online Resource 1). There was no relationship between U.S. citizenship and mental health among White individuals.

Results including length of U.S. residence as a covariate in the overall and race and ethnicity-stratified models (Online Resource 2) were substantively unchanged from those in the original analyses. Additional models limited to foreign-born incarcerated individuals demonstrated that length of U.S. residence was associated with elevated odds of depression and anxiety, but only for incarcerated foreign-born Latino persons (Online Resource 3).

## Discussion

The present study found that U.S. birth was associated with disadvantaged mental health in the overall sample of incarcerated individuals, providing support for the immigrant health advantage. Further analyses revealed that this pattern was only detected among incarcerated Latino persons, which parallels results of a mental health advantage among foreign-born Latino individuals in the general population [16]. The health-eroding effect of living in the U.S. longer was similarly only seen among Latino individuals. These findings indicate that the Latino sample was driving the observed relationships for the overall incarcerated sample, a pattern which is also consistent with previous research [17]. A unique constellation of factors may have contributed to incarcerated Latino immigrants’ mental health advantage in the present sample, including shorter average length of residency in the U.S. compared to other immigrants and higher rates of marriage compared to their U.S.-born counterparts.

Conversely, the immigrant health advantage did not extend to incarcerated Black individuals. While research indicates foreign-born Black individuals tend to display better physical health than U.S.-born Black individuals, evidence of a mental health advantage among Black immigrants is less clear [1, 18]. These mixed findings may be due to differences in mental health outcomes examined, heterogeneity within the Black immigrant population, and/or overrepresentation of Black individuals in confinement, resulting in their exclusion from national surveys [19]. This study is the first to reveal that incarcerated Black immigrants experience increased psychological distress compared to their U.S.-born counterparts. This finding points to an unmet mental health need among incarcerated Black immigrants given their marginalization by systems of race and ethnicity, immigration, and incarceration—factors that are associated with insufficient, low-quality access to mental health services [9, 20, 21].

There was no association between immigration status and mental health for incarcerated White adults. While this finding contrasts with previous research pointing to an immigrant health advantage in mental health among White individuals in the non-incarcerated population [3], it is consistent with the theory that there are racial and ethnic differences in health selection into imprisonment [22]. Since Black and Latino individuals experience disproportionately high incarceration rates, a greater proportion of healthy Black and Latino persons may experience imprisonment relative to White persons. In contrast, with much lower incarceration rates, incarcerated White individuals may be less like non-institutionalized White individuals who have a higher socioeconomic status. Therefore, immigrant health patterns may differ within the incarcerated population by race and/or ethnicity due to differential health selection into prison.

Overall, findings demonstrated that the immigrant health advantage in mental health did not extend to all immigrant populations. The disputed validity of mental health assessments such as the Kessler six-item scale [23], which were primarily developed among White individuals [21], may be contributing to varied findings by race and/or ethnicity. Individuals may conceptualize mental health differently depending on their background [20], meaning traditional assessments of mental health may not be directly applicable to the unique stressors faced by minoritized racial and ethnic groups, incarcerated individuals, immigrants, and other marginalized populations [21].

Our mixed findings by race and/or ethnicity may also be due to varied experiences embedded in the social environment. One of the central ways that structural factors manifest in U.S. society is through inequities in incarceration that disproportionately impact Black and/or Latino individuals [6, 24]. In turn, incarceration removes support networks [25]—which has been proposed as a mechanism underlying the immigrant health advantage [5]—both upon institutionalization and after release. For example, since many immigrants tend to avoid law enforcement due to fear of deportation [26], they may also avoid immigrants who experience incarceration, thereby stripping immigrants of social support and potentially worsening their health [27]. However, these explanations do not explain the findings for incarcerated Latino individuals. More research is needed to understand if systematic differences in mental health conceptualizations, social support, and stigmatization are driving the observed mental health patterns by immigration status and race and ethnicity.

We acknowledge several limitations of this study. The Black and White incarcerated immigrant samples were relatively small, possibly reducing the statistical power to determine significant differences in mental health. We were also unable to disaggregate incarcerated immigrants by country of origin, which may have masked heterogeneity in mental health within the Latino [3, 16], Black, and White immigrant populations. In addition, the study was cross-sectional, meaning we could not address health selection into prison despite evidence demonstrating that some mental health disorders often predate incarceration [9]. Lastly, we were unable to account for length of imprisonment due to the suppression of this information in the public use dataset. This omission may have obscured critical patterns given that incarceration length has been associated with mental health symptoms and likelihood of depression [10] and longer periods of detention can erode health over time [11]. Future studies should consider these nuances in ethnic heterogeneity, health selection, and imprisonment length when examining relationships between incarceration, immigration status, and mental health.

Despite these limitations, the present study highlights previously unexplored disparities in health. The lack of inclusion of marginalized groups in social surveys and their overrepresentation among the incarcerated population [6] underscores an urgent need to better understand existing inequities. Future research should continue to explore whether prevailing hypotheses for the immigrant health advantage are applicable to non-Latino immigrants experiencing disparate social conditions, such as incarceration.

### New Contributions to the Literature

We present novel findings on the mental health of immigrants who are incarcerated. We add to the relatively limited research on the health of Black and White immigrants and extend the immigrant health literature by examining mental health among the incarcerated population. The present study highlights the need to assess health at the intersection of multiple social statuses to reveal the health needs of individuals who are marginalized by systems of incarceration, immigration, and race and ethnicity.

## Supplementary Information

Below is the link to the electronic supplementary material.

Supplementary Material 1

## Data Availability

The Survey of Prison Inmates public-use data can be accessed online through the University of Michigan Inter-University Consortium for Political and Social Research (ICPSR) using the following link: https://doi.org/10.3886/ICPSR37692.v5.
