# Supplementary material for: The Mental Health of Incarcerated Immigrants, Survey of Prison Inmates, 2016
Source: J Immigr Minor Health. 2025 Jul 29;27(5):734–42. doi: 10.1007/s10903-025-01749-z (PMC12420754; doi:10.1007/s10903-025-01749-z)
Supplement: Supplementary file 1 — Supplementary Material 1 [file 10903_2025_1749_MOESM1_ESM.docx]

| **Online Resource 1. U.S. Citizenship and Mental Health Outcomes Regression Models, Overall and Stratified by Race and/or Ethnicity, 2016 Survey of Prison Inmates** | | | | | | |
| --- | --- | --- | --- | --- | --- | --- |
|  | **Model 1: Psychological Distress** | | **Model 2: Depression** | | **Model 3: Anxiety** | |
|  | Coefficient | [95% C.I.] | Odds ratio | [95% C.I.] | Odds ratio | [95% C.I.] |
| **a. Total** (n=20,226) |  |  |  |  |  |  |
| U.S. citizen | 1.30*** | [0.80, 1.79] | 2.73*** | [2.09, 3.56] | 4.15*** | [3.04, 5.67] |
| Female | 1.44*** | [1.13, 1.75] | 2.63*** | [2.30, 3.00] | 3.01*** | [2.65, 3.42] |
| Age (ref=18-34 years) |  |  |  |  |  |  |
| 35-49 years | -0.07 | [-0.29, 0.14] | 1.07 | [0.96, 1.18] | 1.00 | [0.91, 1.11] |
| 50+ years | -0.63*** | [-0.93, -0.33] | 0.82** | [0.72, 0.94] | 0.68*** | [0.60, 0.78] |
| Education (ref=<HS) |  |  |  |  |  |  |
| High school | -0.50*** | [-0.70, -0.30] | 0.79*** | [0.71, 0.89] | 0.90 | [0.80, 1.02] |
| Some college | -0.46** | [-0.74, -0.18] | 0.93 | [0.80, 1.09] | 1.05 | [0.93, 1.20] |
| College degree or more | -0.59* | [-1.04, -0.14] | 0.97 | [0.78, 1.20] | 1.01 | [0.79, 1.30] |
| Race/Ethnicity (ref=White) |  |  |  |  |  |  |
| Black | -0.55*** | [-0.78, -0.32] | 0.47*** | [0.42, 0.52] | 0.35*** | [0.31, 0.40] |
| Latino | -0.71*** | [-1.04, -0.38] | 0.59*** | [0.52, 0.68] | 0.65*** | [0.56, 0.75] |
| Married | -0.01 | [-0.26, 0.24] | 0.94 | [0.83, 1.05] | 1.04 | [0.93, 1.17] |
| Health insurance | -0.85*** | [-1.04, -0.67] | 0.73*** | [0.67, 0.80] | 0.80*** | [0.72, 0.88] |
| Crime type (ref=violent) |  |  |  |  |  |  |
| Property | -0.31* | [-0.59, -0.03] | 0.95 | [0.84, 1.07] | 1.09 | [0.95, 1.26] |
| Drug | -1.11*** | [-1.43, -0.79] | 0.63*** | [0.55, 0.72] | 0.78*** | [0.69, 0.89] |
| Public order | -0.71*** | [-0.96, -0.45] | 0.91 | [0.81, 1.02] | 1.08 | [0.94, 1.24] |
| **b. Black** (n=7,104) |  |  |  |  |  |  |
| U.S. citizen | -1.85 | [-3.76, 0.06] | 0.92 | [0.43, 1.94] | 1.10 | [0.37, 3.30] |
| Female | 1.68*** | [1.23, 2.12] | 2.94*** | [2.37, 3.65] | 3.13*** | [2.47, 3.98] |
| Age (ref=18-34 years) |  |  |  |  |  |  |
| 35-49 years | -0.07 | [-0.39, 0.25] | 1.14 | [0.97, 1.34] | 1.28** | [1.07, 1.52] |
| 50+ years | -0.84*** | [-1.25, -0.44] | 1.01 | [0.82, 1.24] | 0.94 | [0.74, 1.19] |
| Education (ref=<HS) |  |  |  |  |  |  |
| High school | -0.64*** | [-0.96, -0.32] | 0.61*** | [0.51, 0.75] | 0.78* | [0.63, 0.97] |
| Some college | -0.92*** | [-1.35, -0.48] | 0.56*** | [0.43, 0.72] | 0.74 | [0.54, 1.01] |
| College degree or more | -1.31** | [-2.09, -0.53] | 0.61 | [0.32, 1.16] | 0.78 | [0.36, 1.69] |
| Married | -0.11 | [-0.54, 0.32] | 1.25* | [1.01, 1.56] | 1.13 | [0.89, 1.44] |
| Health insurance | -0.89*** | [-1.16, -0.62] | 0.76** | [0.64, 0.90] | 0.77* | [0.63, 0.95] |
| Crime type (ref=violent) |  |  |  |  |  |  |
| Property | -0.19 | [-0.67, 0.28] | 1.10 | [0.90, 1.34] | 1.32* | [1.03, 1.70] |
| Drug | -1.06*** | [-1.44, -0.69] | 0.64*** | [0.52, 0.79] | 0.63*** | [0.49, 0.81] |
| Public order | -0.48* | [-0.91, -0.05] | 0.98 | [0.77, 1.25] | 1.31 | [0.97, 1.77] |
| **c. Latino** (n=5,029) |  |  |  |  |  |  |
| U.S. citizen | 1.62*** | [1.11, 2.13] | 3.30*** | [2.43, 4.50] | 5.64*** | [3.89, 8.17] |
| Female | 2.09*** | [1.44, 2.73] | 2.95*** | [2.29, 3.79] | 3.34*** | [2.66, 4.19] |
| Age (ref=18-34 years) |  |  |  |  |  |  |
| 35-49 years | -0.17 | [-0.65, 0.31] | 0.99 | [0.83, 1.19] | 0.93 | [0.75, 1.14] |
| 50+ years | -0.17 | [-0.79, 0.45] | 0.89 | [0.67, 1.18] | 0.69* | [0.50, 0.96] |
| Education (ref=<HS) |  |  |  |  |  |  |
| High school | -0.45* | [-0.89, -0.01] | 0.91 | [0.73, 1.14] | 0.88 | [0.70, 1.11] |
| Some college | -0.07 | [-0.72, 0.59] | 1.24 | [0.91, 1.67] | 1.03 | [0.74, 1.43] |
| College degree or more | -0.78 | [-1.70, 0.15] | 1.20 | [0.76, 1.89] | 1.53 | [0.97, 2.42] |
| Married | 0.16 | [-0.32, 0.64] | 0.93 | [0.72, 1.20] | 1.12 | [0.89, 1.42] |
| Health insurance | -0.76*** | [-1.18, -0.34] | 0.73** | [0.59, 0.91] | 0.99 | [0.80, 1.22] |
| Crime type (ref=violent) |  |  |  |  |  |  |
| Property | -0.13 | [-0.80, 0.53] | 0.99 | [0.75, 1.31] | 1.08 | [0.79, 1.48] |
| Drug | -0.85** | [-1.41, -0.29] | 0.55*** | [0.43, 0.71] | 0.82 | [0.62, 1.09] |
| Public order | -0.93*** | [-1.44, -0.42] | 0.81 | [0.62, 1.06] | 1.05 | [0.79, 1.40] |
| **d. White** (n=8,093) |  |  |  |  |  |  |
| U.S. citizen | -0.04 | [-1.73, 1.66] | 0.76 | [0.39, 1.48] | 0.94 | [0.45, 1.95] |
| Female | 1.10*** | [0.70, 1.50] | 2.40*** | [2.03, 2.85] | 2.81*** | [2.40, 3.30] |
| Age (ref=18-34 years) |  |  |  |  |  |  |
| 35-49 years | -0.03 | [-0.38, 0.32] | 1.03 | [0.89, 1.20] | 0.90 | [0.77, 1.06] |
| 50+ years | -0.69** | [-1.11, -0.27] | 0.69*** | [0.58, 0.83] | 0.58*** | [0.49, 0.68] |
| Education (ref=<HS) |  |  |  |  |  |  |
| High school | -0.44* | [-0.80, -0.07] | 0.87 | [0.73, 1.04] | 0.97 | [0.82, 1.15] |
| Some college | -0.34 | [-0.77, 0.09] | 1.08 | [0.85, 1.36] | 1.21* | [1.02, 1.45] |
| College degree or more | -0.30 | [-0.87, 0.27] | 1.11 | [0.84, 1.45] | 1.04 | [0.76, 1.40] |
| Married | -0.02 | [-0.40, 0.37] | 0.79** | [0.67, 0.93] | 0.96 | [0.82, 1.14] |
| Health insurance | -0.89*** | [-1.20, -0.58] | 0.71*** | [0.62, 0.82] | 0.73*** | [0.63, 0.85] |
| Crime type (ref=violent) |  |  |  |  |  |  |
| Property | -0.50** | [-0.88, -0.13] | 0.87 | [0.73, 1.03] | 1.01 | [0.84, 1.21] |
| Drug | -1.38*** | [-1.93, -0.84] | 0.65** | [0.51, 0.84] | 0.84 | [0.70, 1.03] |
| Public order | -0.73** | [-1.15, -0.32] | 0.93 | [0.77, 1.11] | 1.01 | [0.85, 1.19] |
|  |  |  |  |  |  |  |
| Notes: Author’s calculations using data from the 2016 Survey of Prison Inmates. Weighted statistics from linear regression (Model 1) and logistic regression (Models 2 and 3). *p<.05; **p<.01; ***p<.001 | | | | | | |

| **Online Resource 2. Immigration Status, Length of U.S. Residence, and Mental Health Outcomes Regression Models, Overall and Stratified by Race and/or Ethnicity, 2016 Survey of Prison Inmates** | | | | | | |
| --- | --- | --- | --- | --- | --- | --- |
|  | **Model 1: Psychological Distress** | | **Model 2: Depression** | | **Model 3: Anxiety** | |
|  | Coefficient | [95% C.I.] | Odds ratio | [95% C.I.] | Odds ratio | [95% C.I.] |
| **a. Total** (n=20,226) |  |  |  |  |  |  |
| U.S.-born | 1.08*** | [0.62, 1.53] | 2.01*** | [1.58, 2.55] | 2.24*** | [1.71, 2.93] |
| Length of U.S. residence | -0.01 | [-0.03, 0.00] | 1.00 | [0.99, 1.01] | 1.00 | [0.99, 1.01] |
| Female | 1.44*** | [1.13, 1.75] | 2.63*** | [2.30, 2.99] | 3.02*** | [2.65, 3.43] |
| Age (ref=18-34 years) |  |  |  |  |  |  |
| 35-49 years | 0.06 | [-0.23, 0.36] | 1.06 | [0.92, 1.22] | 0.98 | [0.85, 1.13] |
| 50+ years | -0.32 | [-0.88, 0.25] | 0.81 | [0.61, 1.08] | 0.66** | [0.50, 0.86] |
| Education (ref=<HS) |  |  |  |  |  |  |
| High school | -0.48*** | [-0.68, -0.28] | 0.80*** | [0.71, 0.90] | 0.91 | [0.81, 1.03] |
| Some college | -0.42** | [-0.70, -0.14] | 0.94 | [0.80, 1.11] | 1.07 | [0.94, 1.22] |
| College degree or more | -0.54* | [-1.00, -0.09] | 0.99 | [0.80, 1.23] | 1.04 | [0.81, 1.33] |
| Race/Ethnicity (ref=White) |  |  |  |  |  |  |
| Black | -0.56*** | [-0.79, -0.33] | 0.47*** | [0.42, 0.52] | 0.35*** | [0.31, 0.40] |
| Latino | -0.75*** | [-1.09, -0.41] | 0.60*** | [0.52, 0.68] | 0.64*** | [0.55, 0.74] |
| Married | -0.04 | [-0.28, 0.21] | 0.93 | [0.83, 1.04] | 1.02 | [0.91, 1.14] |
| Health insurance | -0.84*** | [-1.03, -0.65] | 0.74*** | [0.67, 0.81] | 0.80*** | [0.72, 0.89] |
| Crime type (ref=violent) |  |  |  |  |  |  |
| Property | -0.31* | [-0.59, -0.03] | 0.95 | [0.85, 1.07] | 1.10 | [0.96, 1.27] |
| Drug | -1.14*** | [-1.45, -0.82] | 0.62*** | [0.54, 0.72] | 0.78*** | [0.69, 0.88] |
| Public order | -0.73*** | [-0.99, -0.47] | 0.90 | [0.80, 1.02] | 1.07 | [0.93, 1.22] |
| **b. Black** (n=7,104) |  |  |  |  |  |  |
| U.S.-born | -1.53* | [-2.98, -0.09] | 0.87 | [0.52, 1.46] | 0.79 | [0.35, 1.78] |
| Length of U.S. residence | -0.01 | [-0.04, 0.02] | 1.00 | [0.99, 1.02] | 1.02 | [1.00, 1.04] |
| Female | 1.69*** | [1.25, 2.13] | 2.94*** | [2.37, 3.65] | 3.12*** | [2.46, 3.97] |
| Age (ref=18-34 years) |  |  |  |  |  |  |
| 35-49 years | 0.03 | [-0.44, 0.51] | 1.07 | [0.83, 1.38] | 0.99 | [0.72, 1.38] |
| 50+ years | -0.64 | [-1.53, 0.25] | 0.88 | [0.56, 1.39] | 0.55 | [0.29, 1.04] |
| Education (ref=<HS) |  |  |  |  |  |  |
| High school | -0.64*** | [-0.96, -0.32] | 0.61*** | [0.51, 0.75] | 0.78* | [0.63, 0.97] |
| Some college | -0.93*** | [-1.37, -0.50] | 0.56*** | [0.43, 0.72] | 0.73* | [0.54, 0.99] |
| College degree or more | -1.33** | [-2.11, -0.56] | 0.60 | [0.32, 1.15] | 0.76 | [0.35, 1.65] |
| Married | -0.11 | [-0.54, 0.31] | 1.25* | [1.00, 1.56] | 1.12 | [0.88, 1.43] |
| Health insurance | -0.89*** | [-1.17, -0.62] | 0.76** | [0.64, 0.90] | 0.77* | [0.63, 0.95] |
| Crime type (ref=violent) |  |  |  |  |  |  |
| Property | -0.20 | [-0.67, 0.28] | 1.10 | [0.90, 1.34] | 1.33* | [1.03, 1.71] |
| Drug | -1.06*** | [-1.43, -0.68] | 0.64*** | [0.53, 0.79] | 0.63*** | [0.49, 0.81] |
| Public order | -0.48* | [-0.91, -0.05] | 0.99 | [0.77, 1.25] | 1.32 | [0.97, 1.78] |
| **c. Latino** (n=5,029) |  |  |  |  |  |  |
| U.S.-born | 0.99*** | [0.44, 1.53] | 1.85*** | [1.36, 2.51] | 1.97** | [1.33, 2.92] |
| Length of U.S. residence | 0.02 | [-0.01, 0.04] | 1.02** | [1.01, 1.03] | 1.03** | [1.01, 1.04] |
| Female | 2.08*** | [1.44, 2.73] | 2.91*** | [2.25, 3.76] | 3.32*** | [2.62, 4.21] |
| Age (ref=18-34 years) |  |  |  |  |  |  |
| 35-49 years | -0.35 | [-0.92, 0.22] | 0.79 | [0.62, 1.00] | 0.69* | [0.50, 0.96] |
| 50+ years | -0.52 | [-1.41, 0.36] | 0.54** | [0.34, 0.85] | 0.37** | [0.21, 0.68] |
| Education (ref=<HS) |  |  |  |  |  |  |
| High school | -0.39 | [-0.83, 0.05] | 0.94 | [0.75, 1.18] | 0.92 | [0.73, 1.15] |
| Some college | 0.01 | [-0.64, 0.67] | 1.27 | [0.94, 1.72] | 1.07 | [0.77, 1.50] |
| College degree or more | -0.63 | [-1.54, 0.28] | 1.33 | [0.83, 2.12] | 1.70* | [1.07, 2.71] |
| Married | 0.09 | [-0.39, 0.57] | 0.89 | [0.69, 1.15] | 1.04 | [0.83, 1.31] |
| Health insurance | -0.75*** | [-1.17, -0.34] | 0.73** | [0.59, 0.91] | 1.00 | [0.82, 1.23] |
| Crime type (ref=violent) |  |  |  |  |  |  |
| Property | -0.08 | [-0.75, 0.59] | 1.02 | [0.77, 1.35] | 1.13 | [0.82, 1.55] |
| Drug | -0.83** | [-1.39, -0.26] | 0.57*** | [0.44, 0.73] | 0.83 | [0.63, 1.10] |
| Public order | -0.96*** | [-1.47, -0.45] | 0.81 | [0.62, 1.05] | 1.03 | [0.77, 1.36] |
| **d. White** (n=8,093) |  |  |  |  |  |  |
| U.S.-born | 1.11 | [-0.05, 2.28] | 1.08 | [0.66, 1.77] | 1.41 | [0.80, 2.49] |
| Length of U.S. residence | -0.06*** | [-0.09, -0.03] | 0.98** | [0.96, 0.99] | 0.97*** | [0.96, 0.99] |
| Female | 1.06*** | [0.66, 1.45] | 2.38*** | [2.01, 2.81] | 2.77*** | [2.36, 3.26] |
| Age (ref=18-34 years) |  |  |  |  |  |  |
| 35-49 years | 0.75** | [0.31, 1.19] | 1.36* | [1.07, 1.72] | 1.26* | [1.02, 1.56] |
| 50+ years | 1.11* | [0.20, 2.02] | 1.29 | [0.78, 2.12] | 1.26 | [0.85, 1.87] |
| Education (ref=<HS) |  |  |  |  |  |  |
| High school | -0.42* | [-0.78, -0.06] | 0.87 | [0.73, 1.04] | 0.98 | [0.83, 1.15] |
| Some college | -0.29 | [-0.71, 0.14] | 1.09 | [0.86, 1.39] | 1.24* | [1.05, 1.48] |
| College degree or more | -0.18 | [-0.74, 0.38] | 1.14 | [0.86, 1.50] | 1.08 | [0.80, 1.46] |
| Married | 0.02 | [-0.36, 0.40] | 0.80** | [0.68, 0.94] | 0.98 | [0.83, 1.16] |
| Health insurance | -0.88*** | [-1.19, -0.57] | 0.71*** | [0.62, 0.82] | 0.73*** | [0.63, 0.85] |
| Crime type (ref=violent) |  |  |  |  |  |  |
| Property | -0.57** | [-0.94, -0.20] | 0.85 | [0.72, 1.01] | 0.99 | [0.82, 1.18] |
| Drug | -1.42*** | [-1.95, -0.90] | 0.64** | [0.50, 0.83] | 0.83 | [0.69, 1.01] |
| Public order | -0.78*** | [-1.20, -0.37] | 0.91 | [0.76, 1.09] | 0.99 | [0.84, 1.17] |
| Notes: Author’s calculations using data from the 2016 Survey of Prison Inmates. Weighted statistics from linear regression (Model 1) and logistic regression (Models 2 and 3). *p < .05; **p <.01; ***p <.001 | | | | | | |

| **Online Resource 3. Length of U.S. Residence and Mental Health Outcomes Regression among Foreign-born, Overall and Stratified by Race and/or Ethnicity, 2016 Survey of Prison Inmates** | | | | | | |
| --- | --- | --- | --- | --- | --- | --- |
|  | **Model 1: Psychological Distress** | | **Model 2: Depression** | | **Model 3: Anxiety** | |
|  | Coefficient | [95% C.I.] | Odds ratio | [95% C.I.] | Odds ratio | [95% C.I.] |
| **a. Total** (n=2,143) |  |  |  |  |  |  |
| Length of U.S. residence | 0.01 | [-0.01, 0.04] | 1.02* | [1.00, 1.03] | 1.03*** | [1.02, 1.05] |
| Female | 2.48*** | [1.53, 3.44] | 2.47*** | [1.55, 3.95] | 1.94** | [1.23, 3.05] |
| Age (ref=18-34 years) |  |  |  |  |  |  |
| 35-49 years | -0.82* | [-1.48, -0.16] | 0.98 | [0.66, 1.46] | 0.69 | [0.44, 1.09] |
| 50+ years | -1.08* | [-2.01, -0.15] | 0.53* | [0.30, 0.93] | 0.46* | [0.24, 0.88] |
| Education (ref=<HS) |  |  |  |  |  |  |
| High school | -0.07 | [-0.76, 0.62] | 1.12 | [0.74, 1.68] | 1.47 | [0.95, 2.27] |
| Some college | -0.19 | [-1.18, 0.80] | 1.11 | [0.63, 1.97] | 1.42 | [0.78, 2.59] |
| College degree or more | 0.20 | [-0.89, 1.29] | 1.04 | [0.50, 2.15] | 1.47 | [0.65, 3.31] |
| Race/Ethnicity (ref=White) |  |  |  |  |  |  |
| Black | 1.46 | [-0.16, 3.07] | 0.40** | [0.21, 0.78] | 0.38* | [0.17, 0.87] |
| Latino | -1.09 | [-2.29, 0.11] | 0.25*** | [0.15, 0.43] | 0.34** | [0.17, 0.65] |
| Married | 0.38 | [-0.29, 1.04] | 0.81 | [0.54, 1.23] | 0.74 | [0.47, 1.16] |
| Health insurance | -0.46 | [-1.12, 0.20] | 0.86 | [0.60, 1.25] | 0.91 | [0.60, 1.39] |
| Crime type (ref=violent) |  |  |  |  |  |  |
| Property | -0.04 | [-1.24, 1.16] | 0.75 | [0.40, 1.41] | 0.52 | [0.23, 1.17] |
| Drug | -1.02** | [-1.73, -0.30] | 0.39*** | [0.25, 0.60] | 0.90 | [0.56, 1.42] |
| Public order | -1.36*** | [-2.03, -0.68] | 0.34*** | [0.22, 0.52] | 0.58* | [0.36, 0.94] |
| **b. Black** (n=151) |  |  |  |  |  |  |
| Length U.S. residence | -0.02 | [-0.13, 0.10] | 0.99 | [0.94, 1.04] | 1.03 | [0.96, 1.10] |
| Female | -2.24 | [-4.65, 0.18] | 0.63 | [0.14, 2.95] | 0.83 | [0.10, 6.77] |
| Age (ref=18-34 years) |  |  |  |  |  |  |
| 35-49 years | -1.59 | [-4.83, 1.65] | 1.89 | [0.51, 6.97] | 0.65 | [0.13, 3.36] |
| 50+ years | -0.95 | [-4.43, 2.54] | 1.48 | [0.32, 6.75] | 1.13 | [0.18, 7.08] |
| Education (ref=<HS) |  |  |  |  |  |  |
| High school | -1.68 | [-4.93, 1.57] | 0.71 | [0.22, 2.28] | 1.25 | [0.30, 5.30] |
| Some college | -3.71** | [-6.03, -1.40] | 0.30 | [0.06, 1.52] | 0.28 | [0.04, 1.69] |
| College degree or more | -1.20 | [-4.59, 2.18] | 0.11 | [0.01, 1.13] | 0.33 | [0.02, 4.34] |
| Married | -0.05 | [-2.17, 2.08] | 0.52 | [0.13, 2.03] | 0.77 | [0.16, 3.61] |
| Health insurance | -1.10 | [-3.34, 1.15] | 0.90 | [0.32, 2.54] | 0.89 | [0.26, 3.12] |
| Crime type (ref=violent) |  |  |  |  |  |  |
| Property | -1.24 | [-4.88, 2.41] | 1.17 | [0.17, 8.00] | 0.84 | [0.07, 9.45] |
| Drug | -2.57 | [-5.24, 0.10] | 0.39 | [0.12, 1.24] | 0.04** | [0.01, 0.38] |
| Public order | -3.62* | [-6.70, -0.55] | 0.21 | [0.04, 1.12] | 0.96 | [0.20, 4.53] |
| **c. Latino** (n=1,834) |  |  |  |  |  |  |
| Length U.S. residence | 0.01 | [-0.01, 0.04] | 1.02** | [1.01, 1.04] | 1.05*** | [1.03, 1.07] |
| Female | 3.09*** | [2.01, 4.17] | 3.11*** | [1.84, 5.26] | 2.03** | [1.19, 3.46] |
| Age (ref=18-34 years) |  |  |  |  |  |  |
| 35-49 years | -0.83* | [-1.52, -0.15] | 0.80 | [0.51, 1.27] | 0.61 | [0.37, 1.03] |
| 50+ years | -1.05* | [-2.05, -0.05] | 0.40** | [0.21, 0.77] | 0.26*** | [0.12, 0.55] |
| Education (ref=<HS) |  |  |  |  |  |  |
| High school | -0.14 | [-0.86, 0.57] | 0.97 | [0.59, 1.60] | 1.28 | [0.77, 2.13] |
| Some college | 0.43 | [-0.68, 1.53] | 1.11 | [0.52, 2.37] | 1.29 | [0.63, 2.65] |
| College degree or more | -0.59 | [-1.73, 0.55] | 2.02 | [0.87, 4.70] | 3.34** | [1.45, 7.68] |
| Married | 0.54 | [-0.17, 1.25] | 0.91 | [0.57, 1.45] | 0.78 | [0.47, 1.31] |
| Health insurance | -0.31 | [-1.02, 0.40] | 0.75 | [0.47, 1.20] | 0.89 | [0.55, 1.46] |
| Crime type (ref=violent) |  |  |  |  |  |  |
| Property | 0.60 | [-0.80, 2.01] | 1.00 | [0.48, 2.11] | 0.67 | [0.25, 1.80] |
| Drug | -0.91* | [-1.66, -0.17] | 0.37*** | [0.23, 0.61] | 0.88 | [0.54, 1.44] |
| Public order | -1.25** | [-1.97, -0.53] | 0.33*** | [0.20, 0.54] | 0.57* | [0.33, 0.99] |
| **d. White** (n=158) |  |  |  |  |  |  |
| Length U.S. residence | -0.02 | [-0.09, 0.06] | 1.00 | [0.96, 1.04] | 0.99 | [0.95, 1.03] |
| Female | 1.40 | [-1.00, 3.80] | 0.95 | [0.30, 2.97] | 1.05 | [0.29, 3.80] |
| Age (ref=18-34 years) |  |  |  |  |  |  |
| 35-49 years | 0.04 | [-2.47, 2.54] | 2.61 | [0.80, 8.51] | 1.09 | [0.30, 3.96] |
| 50+ years | -0.81 | [-4.27, 2.66] | 0.94 | [0.22, 4.00] | 1.74 | [0.36, 8.36] |
| Education (ref=<HS) |  |  |  |  |  |  |
| High school | 2.55 | [-0.29, 5.38] | 2.29 | [0.67, 7.88] | 2.01 | [0.52, 7.78] |
| Some college | 2.47 | [-0.49, 5.42] | 2.76 | [0.76, 10.10] | 2.89 | [0.73, 11.42] |
| College degree or more | 5.09** | [1.91, 8.27] | 0.96 | [0.23, 3.98] | 0.69 | [0.14, 3.45] |
| Married | -1.38 | [-4.04, 1.28] | 0.48 | [0.15, 1.53] | 0.54 | [0.16, 1.85] |
| Health insurance | -1.14 | [-3.42, 1.13] | 1.42 | [0.57, 3.57] | 0.78 | [0.28, 2.16] |
| Crime type (ref=violent) |  |  |  |  |  |  |
| Property | -1.12 | [-4.03, 1.79] | 0.67 | [0.21, 2.15] | 0.57 | [0.13, 2.40] |
| Drug | 0.01 | [-3.63, 3.64] | 0.56 | [0.14, 2.25] | 2.31 | [0.50, 10.61] |
| Public order | -0.64 | [-2.93, 1.65] | 0.53 | [0.18, 1.51] | 0.49 | [0.13, 1.75] |
| Notes: Author’s calculations using data from the 2016 Survey of Prison Inmates. Weighted statistics from linear regression (Model 1) and logistic regression (Models 2 and 3). *p < .05; **p <.01; ***p <.001 | | | | | | |
